# Nursing-led education and its impact on knowledge, self-care practices, and pain intensity in women with endometriosis

**DOI:** 10.3389/fpubh.2026.1795899

**Published:** 2026-04-16

**Authors:** Hajer I. Motakef, Salam Bani Hani, Emran A. Abu Aqoulah, Niven Basyouni, Layla Alshammari, Soha Kamel Mosbah Mahmoud, Bahia Galal Abd Elrazik Siam, Elham Saeed Moqbel, Zainh I. Motakef, Fatima Diab, Mokhtar A. Almoliky, Isis Emile Gohar

**Affiliations:** 1 Department of Maternal and Child Health, College of Nursing, University of Hail, Hail, Saudi Arabia; 2 Nursing Department, Irbid National University, Hail, Saudi Arabia; 3 Department of Obstetrics & Gynecologic Nursing, Faculty of Nursing, Alexandria University, Alexandria, Egypt; 4 Department of Medical Surgical Nursing, College of Nursing, University of Hail, Hail, Saudi Arabia; 5 Department of Community Health Nursing, College of Nursing, Hail University, Hail, Saudi Arabia; 6 Department of Nursing, Faculty of Medicine and Health Sciences, University of Hodeida, Hodeida, Yemen

**Keywords:** nursing-led education, knowledge, pain intensity, self-care practices, endometriosis

## Abstract

**Objective:**

Evaluate how a nursing-led education affects knowledge, self-care practices, and pain intensity in women with endometriosis.

**Methods:**

A quasi-experimental study was utilized in this research. The study included 90 women diagnosed with endometriosis who were allocated to either a study group (*n* = 45) or a control group (*n* = 45). Data were collected using a structured knowledge questionnaire, a self-care behaviors scale, and the Visual Analog Scale (VAS) for pain assessment. Measurements were conducted at baseline, post-intervention, and follow-up. Independent *t*-tests, ANOVA, and correlation analyses were used for data analysis.

**Results:**

The research study had a total of 90 women with endometriosis, with a mean age of 32.8 ± 4.95 years, mostly married (58.3%), living in urban areas (74.4%), and with regular menstrual cycles (66.7%). There were no significant differences in baseline knowledge, self-care, and pain intensity between the control and research groups. The research group had higher knowledge and self-care scores compared to the control group after the intervention (*p* < 0.001). In addition, the research group had less pain intensity at the second and third measurements compared to the control group (*p* = 0.002 and *p* < 0.001).

**Conclusion:**

Nursing-led educational interventions effectively enhance knowledge and self-care practices among women with endometriosis and contribute to meaningful improvements in pain intensity. Integrating structured nursing education into routine care may improve disease management and quality of life for affected women.

## Introduction

Endometriosis is a long-lasting gynecological disease. It occurs when endometrium-like tissue grows outside the uterus. This condition relies on estrogen and can cause inflammation, adhesion formation, and subsequent scarring (1).

Moreover, the estimated incidence of endometriosis in women of reproductive age is between 2 and 17%. This condition affects 190 million women around the globe (2, 3). The wide variation in clinical presentation and the complexities of diagnosis lead to discrepancies in reported prevalence and incidence rates (4).

The exact cause of endometriosis is still unclear. Biological, genetic, or environmental factors are suspected to be involved in the development of endometriosis. Biologically, retrograde menstruation, hormonal imbalances, and immune system dysregulation have been suspected to facilitate the implantation and persistence of endometrial tissue outside the uterus. Meanwhile, genetic predisposition is also thought to influence the risk of developing endometriosis, where studies have identified familial clustering and specific gene variants associated with increased susceptibility. Environmental factors including exposure to endocrine-disrupting chemicals, and lifestyle influences are suspected to be further modulate disease risk. Collectively, these factors underscore the complexity of endometriosis pathogenesis (3, 5, 6).

Endometriosis may not cause symptoms in some women, but for many, it leads to painful periods, pain during intercourse, bowel or urinary discomfort, and chronic pelvic pain (7). In some cases, when other organs are affected, symptoms such as cyclic bleeding or shortness of breath may occur (8). These manifestations result from ectopic endometrial tissue, which induces ongoing inflammation, fibrosis, and adhesion formation, underlying the pain and functional impairments experienced by many women (3, 9).

Recent evidence shows that endometriosis is often diagnosed late, with women experiencing an average delay of 7–10 years from symptom onset to definitive diagnosis. This delay is primarily attributed to limited awareness among both women and healthcare providers, as symptoms are frequently downplayed or ignored, leading to postponed treatment and less effective disease management (10–13). Notably, recent evidence suggests that this diagnostic delay has been gradually decreasing in recent years (14). Beyond the clinical impact, endometriosis places a substantial burden on women and their families, affecting physical, psychological, social, and economic well-being. It disrupts daily life, work, education, relationships, fertility, and emotional health, while also generating high costs for healthcare systems and society through increased medical expenses and lost productivity (15, 16).

Moreover, endometriosis also imposes a substantial burden on healthcare systems in 2022–2023, approximately 44,200 hospitalizations, and over 4,800 women with endometriosis who required emergency medical care, with the highest rates occurring among women aged 15–44 years. These encounters highlight the critical and expanding role of nurses in the comprehensive management of endometriosis, including clinical assessment, pain management, patient education, and care coordination. Consequently, strengthening nurses’ knowledge and awareness of endometriosis is essential to enhance the quality of care, support timely recognition and management of symptoms, reduce diagnostic delays, and promote patient-centered care (17).

Although detailed national hospitalization and emergency department data for endometriosis are limited in Gulf and Arab countries, studies indicate that the condition is a significant clinical issue in the region. In particular, endometriosis had been diagnosed in around 10–13% of women presenting for laparoscopic gynecological surgery in Jordan and other Middle Eastern populations, suggesting a substantial healthcare burden and frequent clinical encounters related to symptom management and surgical care (18).

Additionally, research in Saudi Arabia has revealed gaps in nursing knowledge regarding endometriosis, underscoring the importance of enhanced nursing education and support to optimize patient outcomes and reduce delays in diagnosis and management (19).

Therefore, improving knowledge about endometriosis is crucial both at the time of initial diagnosis and for women who are already living with this chronic condition. For newly diagnosed women, increased awareness facilitates early recognition, timely reporting of symptoms, and more accurate diagnosis. For those already diagnosed, better understanding of self-care practices, symptom management, and when to seek medical attention enhances daily coping and reduces reliance on emergency care (20).

Similarly, when healthcare professionals are well-informed, they can provide more effective education, guidance, and care coordination, which ultimately improve overall outcomes. In contrast, poor knowledge or misconceptions whether among women or healthcare providers contribute to misdiagnosis, inadequate management, and ongoing suffering, as evidenced in studies involving women and nurses in diverse clinical settings (20).

Women with endometriosis often express a strong need for early education on managing symptoms and improving their quality of life (21, 22). Enhancing awareness empowers women to advocate for themselves, supports timely care, and enables more personalized management. Alongside medical treatment, self-care strategies such as rest, heat therapy, physical activity, and dietary adjustments, play a vital role in reducing pain intensity, psychological distress, and reliance on medications, making self-care a key component of comprehensive and sustainable endometriosis management. Hence, this study aims to evaluate the effect of the nursing-led education program on knowledge, self-care practices, and pain intensity in women with endometriosis. The study hypothesized that (H1) Women with endometriosis will demonstrate a significant increase in knowledge scores after receiving the nursing-led educational intervention compared to pre-intervention scores, (H2) Women with endometriosis will demonstrate significantly improved self-care practices after receiving the nursing-led educational intervention compared to pre-intervention practices, and (H3) Women with endometriosis who receive the nursing-led educational intervention will report a significantly greater reduction in pain intensity after receiving the nursing-led educational intervention compared to pre-intervention scores.

## Materials and methods

### Design

A quasi-experimental research design was utilized in this study.

### Settings and sampling

This study was conducted at the Gynecology outpatient clinics of El-Shatby Maternity University Hospital. This setting was chosen because it is the only main university hospital in Alexandria Governorate that provides obstetrics and gynecology services as well, and the high follow-up turnover of the women is suitable for the study. Additionally, women attending this hospital had nearly the same socioeconomic status, which preserved the homogeneity of the study sample.

The study included a purposive sample of 90 women diagnosed with endometriosis who attended the gynecology clinic during the data collection period. The participants were chosen if they were aged 18–45 years, experiencing pelvic pain related to endometriosis, able to read and understand the study language, and willing to participate and provide informed consent. However, if the women had been diagnosed with other chronic gynecological conditions causing pelvic pain, were pregnant, or had previously received structured education on endometriosis self-care, they were excluded from the study.

The priori sample size estimation was performed using G*Power software version 3.1 to ascertain the minimum number of participants required to identify differences between the study and control groups. Using a medium effect size, i.e., a value of 0.5 for Cohen’s *d*, a 0.05 alpha level, and a 0.80 statistical power level for a two-tailed independent samples *t*-test, it was estimated that a minimum of 64 participants were required. However, to increase the study’s statistical power, a decision was made to increase the number of participants to 90. The selected women were equally assigned to two groups, i.e., a study group of 45 participants who were provided with a nursing-led educational program, and a control group of 45 participants who were provided with routine care.

### Instrument

The study employed four instruments to collect the required data. The first was a structured socio-demographic and women’s history interview schedule, which included information on age, marital status, occupation, educational level, and residence. It also covered women’s reproductive history, including menstrual, obstetric, and endometriosis-related history. Regarding *regular menstruation*, is defined as the menstrual cycle occurring at consistent intervals ranging from 21 to 35 days, which is considered the normal average cycle length in reproductive-aged women. The second is an Assessment of Women’s Level of Knowledge regarding endometriosis, which includes definitions, risk factors, causes, common sites, manifestations, complications, diagnosis, and treatment of endometriosis. These questions assessed women’s knowledge of endometriosis at baseline (pre-test), 1 month post-intervention (post-test), and 3 months later (follow-up). Each item of the level of knowledge was given a score; correct and complete answers were scored as 2, correct and incomplete answers were scored as 1, whereas incorrect or do not know were scored as 0. The total knowledge score was calculated by the summation of the scores for the” known items, then the scores were converted into percent. The higher scores reflected a higher level of knowledge regarding endometriosis. The total knowledge score was indicated as follows: Good: >75% (≥12), Fair: 75–50% (8 to <12), and Poor: 50% (<8). The third tool was designed to measure the self-care practices in managing endometriosis symptoms. The tool was modified by the researchers based on a comprehensive review of the literature on endometriosis management and non-pharmacological symptom relief strategies. It used to measure the self-care practices of women in relieving symptoms of endometriosis, such as dysmenorrhea, dyspareunia, dysuria, dyschezia, and pelvic pain. The tool was composed of several items that were grouped into five domains: breathing exercises and meditation, heat application (hot liquids and hot compresses), dietary practices, acupressure techniques (Hoku point massage and CV3/CV4 massage), and rest and stretches (23–26). The respondents indicated how often they practiced the self-care activities in relieving symptoms of endometriosis through a three-point Likert scale (1 = never, 2 = sometimes, 3 = always). The internal consistency reliability of the knowledge questionnaire was assessed using Cronbach’s alpha, which demonstrated good reliability in the current sample (*α* = 0.88).

The total score was used to measure the level of self-care practices. The tool was administered before the intervention (pre-test), 1 month after the intervention (post-test), and 3 months after the intervention (follow-up).

The fourth scale was the Visual Analog Scale (VAS), that developed by Katz and Melzack (27). It was adopted and used to assess the current pain intensity of endometriosis-related symptoms such as dysmenorrhea, dyspareunia, dysuria, dyschezia, and pelvic pain. The VAS represented a self-report device that consists of a horizontal line used for subjective estimation of the patient’s pain. It comprised a 10-point numerical scale, corresponding to the degree of pain intensity, with zero representing no pain and 10 representing the unbearable pain. In between these two opposite ends, words as mild, moderate, and severe are assigned to each 3 cm distance, respectively. Women were asked to place a mark on the line at the point that represents the pain intensity. The total score ranged from 0 to 10 as follows: No pain = 0, Mild pain = 1–3, Moderate pain = 4–6, Severe pain = 7–9, and Unbearable pain = 10.

### Data collection method

The study was conducted through a series of systematic steps. Ethical approval was obtained from the Ethical Committee of the Faculty of Nursing, Alexandria University (AU-20-3-255). Subsequently, an official letter was issued from the Faculty of Nursing to the responsible authorities at the study settings to obtain permission for data collection after clearly explaining the study purpose. The knowledge questionnaire was created by the researcher based on her review of relevant literature. The content validity was determined by a panel of five experts in obstetric nursing, gynecologic nursing, and women’s health. The content validity index was between 0.80 and 1.00 for items, while the scale was 0.92. The self-care practices scale was based on previous studies on symptom management for endometriosis. The scale was translated into Arabic using forward and backward translation. The two scales were reviewed by a panel of experts. The reliability of the scales was determined by Cronbach’s alpha. The alpha was good for both the knowledge questionnaire (0.88) and the self-care scale (0.86). The study was piloted on nine women to ensure clarity. The wording was slightly changed based on feedback.

Eligible women with endometriosis were selected using purposive sampling from the gynecology outpatient clinic according to the predetermined criteria. The selected women were then randomly assigned to two equal groups. The random assignment was done using a simple randomization method with the aid of random numbers. The sample consisted of 90 women, with 45 women forming the control group and the other 45 forming the study group. The control group received routine care for endometriosis at the hospital. The women were also administered the study tools at three different time periods: before routine care, after 1 month, and after 3 months. To avoid the possibility of the participants sharing information, the data collection for the control group was done before the intervention for the study group. The reason for selecting the control group before the study group was to avoid the sharing of the educational sessions by the participants. The participants were in the same clinical setting, which would have affected the results if the two groups had been selected at the same time. The time between the two phases was short, and the setting was the same. The setting was constant during the study period, thus reducing the chances of confounding. Women in the study group received the educational program provided by the nurses, in addition to the usual hospital care. The women in the study group were assessed before the start of the intervention, a month following the intervention, and 3 months following the intervention, using the same instruments as the control group.

### Ethical considerations

Regarding ethical considerations, written informed consent was obtained from all participating women after explaining the study aim, in addition to a witnessed written consent from the head nurse. Participants’ privacy was ensured, and the confidentiality of all collected data was strictly maintained. Women were informed that participation was voluntary and that they had the right to withdraw from the study at any time without any effect on the care they received at the hospital.

### Data analysis

Following data collection, the data were categorized, coded, tabulated, and computerized. Both descriptive and analytical statistical analyses were performed using the Statistical Package for Social Sciences (SPSS) version 20, with a significance level set at *p* ≤ 0.05. Normality of continuous data was assessed using the Shapiro–Wilk test. Independent *t*-tests and a mixed-design repeated measures ANOVA were used to compare outcomes between and within groups over time. Pearson’s correlation was applied to examine associations between continuous variables, and Chi-square tests for categorical variables. Missing data (<5%) were handled using pairwise deletion. The effect of nursing-led education on knowledge, self-care practices, and pain intensity in women with endometriosis was determined by comparing outcomes between the study and control groups before and after the intervention.

## Results

### Sample characteristics

The study sample comprised 90 women diagnosed with endometriosis, with a mean age of 32.8 years (SD = 4.95). Regarding occupational status, nearly half of the participants were housewives (47.8%), followed by workers (30.0%) and employed women (22.2%). In terms of educational attainment, the largest proportion had completed secondary education (40.0%); a smaller segment of the sample was illiterate (15.6%). Most participants were married (58.3%), whereas 41.7% were single. The majority of women resided in urban areas (74.4%), with the remaining participants living in rural settings (25.6%). Concerning menstrual characteristics, two-thirds of the sample reported regular menstruation (66.7) (Table 1).

**Table 1:** Sample characteristics of women (*n* = 90).

Variables	*N* (%)
Age in years	*M* (SD) 32.8 (4.95)
Occupation	
Housewife	43 (47.8)
Worker	37 (30.0)
Employed	20 (22.2)
Education	
Illiterate	14 (15.6)
Primary	20 (22.2)
Secondary	36 (40.0)
University	20 (22.2)
Marital status	
Single	42 (41.7)
Married	48 (58.3)
Residence	
Urban	76 (74.4)
Rural	23 (25.6)
Regularity of menstruation	
Regular	60 (66.7)
Irregular	28 (33.3)

M, mean; SD, standard deviation; n, number; %, percentage.

### Total level of knowledge among women with endometriosis

There was no significant difference in the level of knowledge between the study and control group at the outset (*p* = 0.430). However, the level of knowledge in the study group increased significantly at the second and third assessments compared to the control group (*t* = −4.53 and −4.82, respectively; *p* < 0.001) (Table 2).

**Table 2:** Comparison of total knowledge levels between study and control groups among women with endometriosis.

Total level of knowledge	Group	*M* (SD)	*T*	df	Sig.	95% CI
Total level of knowledge 1 (Pre-test)	Study (*n* = 45)	6.67 (1.74)	0.793	88	0.430	−0.46 to 1.09
	Control (*n* = 45)	6.98 (1.97)				
Total level of knowledge 2 (Post-test)	Study (*n* = 45)	10.7 (3.00)	−4.53	88	<0.001	−3.74 to −1.45
	Control (*n* = 45)	8.07 (2.42)				
Total level of knowledge 3 (Follow-up)	Study (*n* = 45)	12.3 (3.44)	−4.82	88	<0.001	−4.83to − 2.01
	Control (*n* = 45)	8.87 (3.28)				

df, degree of freedom; Sig. Level of significance at *P* ≤ 0.05; CI, confidence interval.

### Total level of self-care practices among women with endometriosis

No significant difference in self-care practices was observed between groups at baseline (*p* = 0.310). However, the study group demonstrated significantly higher self-care scores at the second and third assessments compared with the control group (*t* = −4.93 and *t* = −4.89, respectively; *p* < 0.001) (Table 3).

**Table 3:** Comparison of total self-care practices between study and control groups among women with endometriosis.

Total level of self-care	Group	*M* (SD)	*t*	df	Sig.	95% CI
Total level of self-care 1 (Pre-test)	Study (*n* = 45)	55.5 (5.99)	−0.206	88	0.310	−2.84 to −2.31
	Control (*n* = 45)	55.2 (6.28)				
Total level of self-care 2 (Post-test)	Study (*n* = 45)	69.1 (11.7)	−4.93	88	<0.001	−15.1 to −6.42
	Control (*n* = 45)	58.3 (8.83)				
Total level of self-care 3 (Follow-up)	Study (*n* = 45)	76.4 (16.5)	−4.89	88	<0.001	−21.1 to −8.89
	Control (*n* = 45)	61.4 (12.2)				

df, degree of freedom; Sig. Level of significance at *P* ≤ 0.05; CI, confidence interval.

### Level of pain intensity among women with endometriosis

At baseline, there was no significant difference between the study group and the control group with regard to pain intensity, as indicated by the results where *t* = −1.15, *p* = 0.25. The pain intensity was significantly lower at the second assessment compared to the control group, as indicated by the results, where *t* = 3.19, *p* = 0.002. The pain intensity was even more significantly lower at the third assessment compared to the control group, as indicated by the results where *t* = 4.93, *p* < 0.001 (Table 4).

**Table 4:** Comparison of total visual analog scale (VAS) between study and control groups among women with endometriosis.

Total VAS level	Group	*M* (SD)	*t*	df	Sig.	95% CI
Total VAS 1 (Pre-test)	Study (*n* = 45)	8.27 (1.34)	−1.15	88	0.25	−9.1 to 0.24
	Control (*n* = 45)	7.93 (1.41)				
Total VAS 2 (Post-test)	Study (*n* = 45)	6.00 (1.86)	3.19	88	0.002	0.43 to 1.84
	Control (*n* = 45)	7.13 (1.49)				
Total VAS 3 (Follow-up)	Study (*n* = 45)	3.84 (2.88)	4.93	88	<0.001	1.61 to 3.77
	Control (*n* = 45)	6.53 (2.24)				

df, degree of freedom; Sig. Level of significance at *P* ≤ 0.05; CI, confidence interval.

### Differences between total knowledge, self-care practices, and total level of pain intensity based on demographic characteristics

Analysis of the associations between demographic characteristics and outcomes among women with endometriosis revealed several significant relationships. Age was significantly positively correlated with pain intensity (VAS) (*r* = 0.267, *p* = 0.011), suggesting that older women experienced higher levels of pain, while no significant correlations were observed between age and total knowledge or self-care practices. Occupational status was significantly associated with pain intensity (*F* = 8.36, *p* = 0.001), but not with knowledge or self-care practices. Similarly, educational level was significantly associated with pain intensity (*F* = 5.69, *p* = 0.001), whereas differences in knowledge and self-care practices did not reach statistical significance.

Marital status was significantly related to self-care practices (*t* = −2.11, *p* = 0.039), with married women demonstrating higher levels of self-care compared with single women, but it was not associated with knowledge or pain intensity. No significant differences were found for residence in relation to any of the outcomes. Finally, the regularity of menstruation was significantly associated with pain intensity (*t* = −3.24, *p* = 0.002), indicating that women with irregular menstrual cycles experienced higher pain levels, while knowledge and self-care practices were unaffected. Overall, pain intensity appeared most sensitive to demographic differences, whereas knowledge and self-care practices were largely independent of most demographic characteristics (Table 5).

**Table 5:** Differences in total knowledge, self-care practices, and pain intensity across demographic characteristics in women with endometriosis.

Variables		Total knowledge	Total self-care practices	Total VAS
Age	Pearson correlation = *r*	−0.091	−0.083	0.267
	Sig. (2-tailed)	0.393	0.439	0.011*
Occupation	*F*-value	2.80	2.72	8.36
	Sig. (*p*-value)	0.066	0.072	0.001*
Education	*F*-value	2.53	2.29	5.69
	Sig. (*p*-value)	0.062	0.085	0.001*
Marital status	*t*-value	0.51	−2.11	0.52
	Sig. (*p*-value)	0.061	0.039*	0.602
Residence	*t*-value	0.45	−0.97	−1.65
	Sig. (*p*-value)	0.66	0.336	0.104
Regularity of Menstruation	*t*-value	−0.17	0.10	−3.24
	Sig. (*p*-value)	0.865	0.919	0.002*

*P-value is significant at ≤0.05.

### Mixed-design repeated measures ANOVA for study

The revised analysis demonstrated that while there were no significant baseline differences between groups (non-significant main effect of group across all outcomes), there were statistically significant effects of time, indicating changes within groups. More importantly, significant time × group interaction effects were observed for total knowledge, total self-care, and pain scores (*p* < 0.001 for all), indicating that the intervention group showed significantly greater improvement over time compared to the control group (Table 6).

**Table 6:** Mixed-design repeated measures ANOVA for study.

Outcome Variable	Effect	*F*(df1, df2)	*p*-value	Partial *η*^2^	95% CI (*η*^2^)
Total knowledge	Time	*F*(2, 140) = 48.72	<0.001	0.410	0.30–0.50
	Group	*F*(1, 70) = 0.30	0.588	0.004	0.00–0.06
	Time × Group	*F*(2, 140) = 26.91	<0.001	0.278	0.18–0.38
Total self-care	Time	*F*(2, 140) = 6.84	0.002	0.089	0.03–0.16
	Group	*F*(1, 70) = 0.01	0.907	0.000	0.00–0.02
	Time × Group	*F*(2, 140) = 19.77	<0.001	0.220	0.12–0.32
Pain (VAS)	Time	*F*(2, 140) = 39.55	<0.001	0.361	0.25–0.46
	Group	*F*(1, 70) = 0.66	0.417	0.009	0.00–0.08
	Time × Group	*F*(2, 140) = 31.84	<0.001	0.313	0.21–0.41

## Discussion

Endometriosis is a chronic gynecological condition often diagnosed late due to limited awareness. Nursing-led education can bridge this gap by improving knowledge, promoting self-care, and supporting effective symptom management. This study aimed to evaluate the impact of a nursing-led educational program on knowledge, self-care practices, and pain intensity among women with endometriosis. In this quasi-experimental study, women who received the educational program showed significant improvements in knowledge and self-care practices, as well as reduced pain intensity, highlighting the value of structured nursing education in enhancing outcomes for women with endometriosis.

The findings of the current study demonstrated that the socio-demographic characteristics of the study and control groups were largely comparable. This homogeneity is expected, as the participants were drawn from similar socioeconomic backgrounds, a trend that has also been reported in recent endometriosis-related intervention studies. Such baseline comparability is methodologically advantageous, as it minimizes the influence of potential confounding variables that could affect participants’ responses to the intervention.

On assessing the effect of the nursing-led program on total knowledge levels, the results of the present study demonstrated that there was no statistically significant difference between the study and control groups at baseline. Although a considerable proportion of participants had secondary education, educational level was not significantly associated with knowledge in the current study. This finding suggests that knowledge gaps may not be solely explained by formal education, but could also be influenced by limited disease awareness, lack of targeted health education, and restricted access to reliable information about endometriosis.

The results of the current study showed highly significant education knowledge scores posttest and follow-up among the study group, compared to the control group. This finding is consistent with a study performed by Mohamed et al. (28), who confirm the positive impact of nursing-led education programs on the level of knowledge about endometriosis. Besides, a quasi-experimental study performed by Alasser et al. (29) reported that most participants had insufficient knowledge of endometriosis before the training sessions. Following the educational intervention, participants’ knowledge significantly improved, with posttest and follow-up scores notably higher than pretest scores.

The present study demonstrated that the implementation of the educational program had a significant positive effect on participants’ self-care practices. The study group exhibited notably higher mean scores than the control group both immediately after the program and at follow-up, compared to their baseline self-care levels. This improvement may be attributed to the participants’ engagement with the program content, which covered areas such as proper diet, exercise, and pain management, thereby enhancing their knowledge and awareness on how to manage and alleviate endometriosis symptoms. These findings are partially consistent with a study conducted by Yousif et al. (30), which reported significant improvements in women’s self-care practices, including dietary habits and exercise, 3 months after receiving health education compared to pre-education levels. Similarly, Mohamed et al. (28) reported the effects of instructional nursing strategies on endometriosis, observed a statistically significant enhancement in women’s self-reported care practices for symptom management following the intervention, and at follow-up.

No significant differences in pain intensity between the groups were noted in the current study before the intervention, indicating similarity between the groups before the intervention. However, a significant reduction in pain score was noted between the study and control groups in the second and third measurements. This implies that the nursing-led educational program had a positive impact on the pain intensity among the women in the study group compared to the control group. Although the findings of the current study showed a significant difference between the groups regarding pain intensity, pain perception is also influenced by a number of factors, such as disease severity and psychological factors, and recommended self-care practices.

This result aligns with previous findings performed by Rahmioglu et al. (31) and Taylor et al. (32), indicating that women with endometriosis frequently experience moderate-to-severe chronic pain, reflecting the persistent and complex nature of the disease. Additionally, at the second assessment point, pain intensity decreased clearly in both groups, with a greater reduction observed in the study group, the between-group difference was statistically significant, this trend suggests a potential beneficial effect of the study intervention. Unsimilar findings have been stated in a previous study performed by Kori et al. (33), where short-term follow-up periods may be insufficient to unveil the statistically significant differences despite noticeable clinical improvement, especially in chronic pain conditions such as endometriosis.

At follow-up period, participants in the study group demonstrated a greater reduction in pain intensity than those in the control group. Although the intervention primarily targeted knowledge and self-care practices through nursing-led education, this improvement can also be interpreted within a biopsychosocial framework. Nursing-led education likely enhanced participants’ understanding of endometriosis, promoted engagement in self-management strategies, and facilitated lifestyle modifications, all of which may contribute to improved pain coping. Evidence from women with chronic pain conditions indicates that psychosocial resources such as social support, self-efficacy, and satisfaction with treatment are associated with better adaptation to pain, whereas higher pain intensity correlates with poorer psychological well-being (34). Studies in women with vulvodynia further emphasize that cognitive-behavioral factors, illness perceptions, fatigue, and psychological distress significantly influence both pain intensity and interference, highlighting the interplay between knowledge, coping, and behavioral strategies in shaping pain experiences (35). Collectively, these findings suggest that interventions enhancing knowledge and self-care may indirectly reduce perceived pain by strengthening psychological and behavioral resources.

In the present study, age was significantly positively correlated with pain intensity, indicating that older women reported higher levels of pain compared to younger participants. The current finding is incompatible with a cohort Analysis conducted by Vuontisjärvi et al. (36) who showed a decreased pain threshold and maximal pain tolerance among women with endometriosis in the late fertile age. This potentially reflects long-term inflammatory, central sensitization, and psychosocial adaptations to chronic pain conditions. These divergent findings indicate that pain perception in endometriosis changes across the lifespan and is shaped by a complex, non-linear, and context-dependent relationship between age, pain subtype, and individual characteristics.

The absence of significant correlations between age and total knowledge or self-care practices in this study suggests that knowledge acquisition and the adoption of self-care practices may be influenced more by educational exposure and intervention engagement than by chronological age alone, emphasizing the importance of tailored educational and self-management support irrespective of age.

The current study found that occupational status was significantly associated with pain intensity, with employed participants reporting lower pain levels. This aligns with biopsychosocial models suggesting that engagement in meaningful work may provide social support, a sense of control, and adaptive coping opportunities, which buffer the intensity of pain. Previous research in women with chronic pain supports this, showing that psychosocial resources are linked to better pain adaptation (34). Additionally, Chisari and Chilcot (35) reported that cognitive-behavioral factors, fatigue, and psychological distress significantly influence pain intensity, highlighting the interplay between psychosocial, behavioral, and occupational factors in modulating pain experiences. Conversely, unemployment or unstable work status may elevate stress, reduce access to care, and result in worse pain through biopsychosocial pathways.

Moreover, the present study revealed that educational level was significantly associated with pain intensity. This result corresponds with a study performed by Chowdhury et al. (37) in Bangladesh. They reported associations between sociodemographic characteristics and clinical features, offering contemporary, real-world data on how factors like education relate to pain. Additionally, marital status was significantly related to self-care practices, with married women demonstrating higher levels of self-care compared with single women. This can be interpreted as meaning that married women may benefit from greater dyadic support, facilitating more structured and engaged self-care compared with single women, who may lack consistent partner support.

The regularity of menstruation was significantly associated with pain intensity, indicating that women with irregular menstrual cycles experienced higher pain levels. This result is congruent with the global symptom surveys performed by Demetriou et al. (38), where irregular bleeding alongside pelvic pain were frequently reported by women with endometriosis, which supports the link between disrupted menstruation and pain burden.

Consequently, nursing-led education is well recognized as a key component of managing chronic diseases including endometriosis. It can enhance patient knowledge, self-care practices, and symptom management. By providing the evidence-based information on early symptoms recognition, pain relief measures, and lifestyle modification, these programs address gaps in awareness, and promote sustained self-management as well (28, 39–41). Therefore, the present study’s findings reinforce the pivotal role of nurses in empowering women with endometriosis through structured, patient-centered education.

This study underscores the importance of nursing-led educational programs in helping women with endometriosis. Such programs enhance knowledge, promote effective self-care, and reduce pain, empowering women to manage their symptoms through lifestyle changes and healthy practices. The findings also suggest that personal factors like age, marital status, education, and menstrual patterns influence pain intensity and self-care practices, highlighting the need for tailored, supportive interventions. Overall, combining education, guidance, and ongoing support can improve symptom management, boost confidence, and enhance quality of life for women living with endometriosis.

## Conclusion

Nursing-led educational program can effectively improve knowledge, enhance self-care practices, and reduce pain intensity in women with endometriosis. These findings highlight the essential role of nurses in patient education and support the integration of structured, ongoing education into routine care to optimize symptom management and long-term outcomes. Nurses play a critical role in empowering women with endometriosis through education that promotes self-care and symptom management.

### Clinical implications

The findings of current study underscore the essential role of nursing-led educational interventions in enhancing knowledge, self-care practices, and pain management among women with endometriosis. By empowering women with accurate information and beneficial self-care practices, nurses can facilitate more effective symptom management, reduce reliance on pharmacological treatments, and improve overall quality of life. Implementing structured education programs within routine gynecological care may also promote woman engagement, early recognition of symptoms, and timely healthcare-seeking behaviors, thereby addressing key public health concerns related to chronic reproductive conditions. These interventions have the potential to be adapted and scaled in community health and primary care settings to reach a broader population of women at risk of delayed diagnosis or suboptimal disease management.

### Limitations

The study also has a number of limitations that should be noted. First, it should be noted that due to the quasi-experimental approach and lack of full randomization, it is possible that there may be limitations to internal validity and a risk of selection bias. It should be noted that a sequential approach to data collection was used to minimize bias between groups, although it is not possible to eliminate the risk of information sharing. Secondly, due to the sampling approach used, it is possible that it may not be fully applicable to other populations. Thirdly, due to the lack of blinding, it is possible that measurement bias may have been a problem. Lastly, it should be noted that the three-month period is a short period to assess the full impact of endometriosis, which is a recurrent condition.

### Research opportunities

Future research should aim to address these limitations by employing multi-center, randomized controlled designs with larger sample sizes to strengthen causal inference and generalizability. Extended follow-up periods are recommended to assess the long-term sustainability of knowledge gains, self-care practices, and pain outcomes. Additionally, studies exploring implementation strategies for scaling nursing-led education interventions in diverse clinical and community settings are warranted. Investigating the integration of digital health tools, tele-education, or culturally tailored programs may further enhance accessibility and effectiveness, supporting broader public health initiatives aimed at improving reproductive health literacy and patient-centered care for women with endometriosis.

## Data Availability

The raw data supporting the conclusions of this article will be made available by the authors, without undue reservation.
